# Who should be responsible for the care of advanced chronic kidney disease? Do the guidelines point to the end of nephrology follow-up of advanced CKD or are they the starting point for a new approach?

**DOI:** 10.1186/s12882-020-01908-4

**Published:** 2020-07-29

**Authors:** Giorgina Barbara Piccoli

**Affiliations:** 1 Department of Clinical and Biological Sciences, University of Torino, 10124 Torino, Italy; 2 Nephrologie, Centre Hospitalier Le Mans, 72037 Le Mans, France

**Keywords:** Chronic kidney disease, Chronic care, Compliance, Primary care physicians, Nephrology, Guidelines, Personalised medicine

## Abstract

The editorial comments on a recently published study in which 242 patients, with “stable” chronic kidney disease, recruited during a hospital stay, were randomised either to receiving support from nephrologists (co-management by primary care physicians and nephrologists), or to be managed by primary care physicians with written instructions and nephrology consultations on demand. After a mean follow-up of 4 years, the results in terms of dialysis start, hospitalisation and death were similar for both groups.

This study gave the possibility to discuss about the options of follow-up of CKD patients, including on one side the advantage of a greater involvement of primary care physicians, who could oversee care by applying a common set of simplified guidelines, and on the other one the importance of a direct and deep involvement of the specialists that seems necessary in particular if personalised approaches have to be pursuit. The data of the present study are somehow in disagreement with the literature, usually suggesting better outcomes in intensive treatment, in which specialists are directly involved. The literature is heterogeneous, the goals vary and the populations are differently selected. The compliance issue is probably one of the missing pieces of the puzzle, and specific interventions should also be tailored to “reluctant” patients. Guidelines should probably be staring points for improvement, and not the standard of care; the study herein discussed may suggest that primary care physicians may be of great help in granting a good standard of care, hopefully as a baseline for further improvement, and personalised care.

## Correspondence

In an elegant randomised controlled study, Saudan and co-workers, at the prestigious University of Geneva, allocated 242 patients, with “stable” chronic kidney disease, recruited during a hospital stay, to one of two groups: one whose follow-up included support from nephrologists (co-management by primary care physicians and nephrologists); the other managed by primary care physicians with written instructions on treatment goals, nephrology consultations are on demand. After a mean follow-up of more than 4 years, the results in terms of dialysis start, hospitalisation and death were similar for both groups [1].

This result is interpreted by the authors as an indication that in the follow-up of CKD patients there should be greater involvement of primary care physicians, who could oversee care by applying a common set of simplified KDOQI guidelines, and that this policy would have a significant positive effect on nephrologists’ workload [1].

Given the dire lack of studies on the management of advanced CKD, the authors should be commended for their ambitious, well-conducted long-term trial.

In the few other studies available, the results are heavily dependent on setting and goals: in the study called MASTERPLAN, an acronym for “Multifactorial Approach and Superior Treatment Efficacy in Renal Patients with the Aid of Nurse Practitioners”, the added intervention of a nurse practitioner was associated with a striking 20% reduction in the composite negative endpoint, albeit not with better cardiovascular outcomes (which is not surprising if we consider that cardiovascular care is probably a more medical task, and that in most cases in advanced CKD “*les jeux –cardiovasculaires- sont faits*”, and reversing the biological clock is probably impossible with our present treatment tools) [2]. A cluster randomised study called ESCORT (Effectiveness of Integrated Care on Delaying Progression of Stage 3–4 Chronic Kidney Disease in Rural Communities of Thailand), which involved 442 CKD patients, once more not surprisingly, demonstrated that integrated care works better than follow-up by only one specialist [3]. In this study the incidence of adverse outcomes was about halved in patients followed with the support of a large multidisciplinary team consisting of two general practitioners, two chronic care nurses, a pharmacist, a nutritionist, a physical therapist, a health care officer, 3 to 5 village health care volunteers, and the family members of CKD patients residing in the area [3].

Interestingly, the ESCORT study is in line with the mother of all battles, the STENO2 study in diabetic patients, published in The New England Journal of Medicine in 2003, which demonstrated, in 160 randomised subjects, the clear superiority of intensive treatment, which consisted in a series of interventions aimed at behaviour modification and a stepwise introduction of pharmacologic therapy overseen by a project team (doctor, nurse, and dietician) at the Steno Diabetes Center [4]. This pivotal Danish study demonstrated a significant advantage for all the tested outcomes in diabetic patients, including two of particular relevance for the nephrology community, i.e. the development of kidney disease and the need for renal replacement therapy [4, 5].

Unlike previous positive results, and in line with a completely different selection of its study population, was a Canadian study aimed at assessing the possible advantages of a nurse-coordinated model of care. The patients enrolled were identified from laboratory records as having a low e-GFR level, but for the most part (96%) had not previously been followed up by a specialist and the study found that this approach did not improve outcomes and resulted in a higher pill burden [6]. The authors conclude that some CKD patients, like those identified through community laboratories, do not have progressive kidney disease and, in such a context, specialized nephrology care cannot make a difference [6].

A similar trial was conducted in the United States with a different selection of patients with stages 4–5 CKD, who were already receiving nephrology care in various settings, and demonstrated an advantage in reducing hospitalisation rates and improving patients’ preparation for renal replacement therapy in the arm with coordinated care [7].

Three recent systematic reviews have also addressed this question. While the first two conclude that data indicate that the involvement of different professionals (nurses, primary care phsydsicians and others) has a positive effect, the third one, while acknowledging the advantages that have been reported, points out the heterogeneity of the studies published in terms of multidisciplinary CKD clinic composition, entry criteria, follow-up, and type of care [8–10].

Given this background, how should we read the negative results of the Swiss study?

A simplistic conclusion is that it is not worth offering nephrology care (and consequently even less important to offer complex integrated care) to patients with advanced CKD, since, with brief, clear, written instructions summarizing the current CKD guidelines, knowledgeable general practitioners perform as well as nephrology specialists, at least for patients who have a “stable” CKD. The paper may therefore seem to be in agreement with those who consider that the guidelines spell the end of specialist care in treatment of this illness [11].

On a second glance, however, our reading of the results could differ significantly.

The paper design is unique, with respect to the other studies cited, in which intervention involves an “add-on” to standard care. The Swiss study’s proposal should instead be seen as a “subtract-from” approach, as the usual nephrology care is absent from one of the two arms [1].

The difference reflects something deeper, philosophically as well as clinically: an add-on approach is chosen when we try to answer the question of what we can do to improve care that we perceive as insufficient, and unsatisfactory. In this regard, it is logical that each study envisions different additions: there are no village health volunteers in North American cities, nor is it likely that there will be specialised managing nurses in remote Thailand villages. Each study adds what is considered to be of added value, within a widespread policy of sustainability. Given the heterogeneity described above, the common objective, expressed in the different backgrounds of the studies, is finding what can be done to integrate services to improve outcomes.

Conversely, the original study commented on, in which the interventional arm offers “less care” than usually given, implicitly considers that patients (i.e. most patients, stable patients, "non-progressive patients …) are already receiving the best care presently available, and that this care is faithfully reflected in the current guidelines [1].

This position is interesting, and outside the box.

At present healthcare systems are increasingly focussing on how to improve care of advanced CKD, France, for example, has established a bundle reimbursement system, which requires that nephrology care be integrated with educational programs and nutritional management. Given this trend, suggesting that there are cases who can safely receive “lighter” care may contribute to clarifying ideas.

The selection of patients is an important issue; interestingly, in the Swiss study, patients with previously known CKD did less well than those who had not received follow-up care. This can be seen as an indication of how well the Swiss system functions, since the logical interpretation is that the patients with poorer prognoses had been already identified and referred. More interestingly, however, of those that theoretically could have been randomised, the patients who declined had worse outcomes. This “self-selection” by patients is probably one of the reasons why the ones who accepted being randomised did better, and it is reasonable to suppose that in the refusal cohort non-adherence was a major negative factor. In this regard, one of the advantages of the trial was to identify a population in which lack of participation was probably the epiphenomenon of a more generalized negative attitude towards care. This is a population that should be systematically identified, and should be encouraged to seek treatment via tailored interventions (education, follow-up reminders, empowerment programs).

In the description of a standard intervention it is not fully clear what “dietician and lifestyle counselling” means, and who provides this in the context of a 30-min consultation. Notably, in no part of the paper, is “standard care” described as involving a team and consisting in coordinated personalised care. While the authors cite as a limitation failure to prescribe low−/very low-protein supplemented diets (very low supplemented diets usually feasible in 10–20% of patients) or strict sodium restriction (rarely recommended in advanced CKD), they leave us with the idea that patients are managed in accordance with the international nutritional guidelines that, at the time of publication of this paper, are twenty years old [11].

As it is, this important paper may convey the message that a good nephrologist is no better than an educated primary care physician at following the KDOQI guidelines. It stresses that, given similar clinical conditions (fulfilling the same enrolment criteria), non-clinical factors (e.g. willingness to participate) make a major difference in patient survival, and indirectly suggests that interventions should also be tailored to “reluctant” patients.

Actually, there is an important missing point: the system of care included treatment advice for the general practitioners, provided by nephrologists on request. Details of the requests, their type and frequency are not provided in the paper, but it is reasonable to think that this distant “maternage” was crucial in the attainment of the results. Indeed, as the recent pandemic has sown, there is a great potential for implementing remote care, for improving, hopefully without replacing, standard one. This could help better coping with the limited number of specialists in many highly resourced countries, as well as the limited number of physicians in resource-limited settings; how to maintain a distant but humane approach to our chronic patients will probably represent our next challenge.

Some nephrologists strongly believe, and I am one of them, that guidelines should be staring points for improvement, and not the standard of care. Guidelines change, and are often already outdated when they first appear [12]. Large, influential, settings, particularly university-affiliated hospitals, should be examples of new approaches to treatment and better care; personalization of treatment, integrated care, and dietary management offer new opportunities and are continuously being updated. The lack of difference in the effect of guideline-driven care, observed in compliant patients with stable CKD in this study, can be read as a good reason to go beyond the guidelines to improve the care of patients with advanced CKD.

## Data Availability

Not required for this editorial paper.

## Abbreviations

CKD: Chronic kidney disease
KDOQI: Kidney Disease Outcomes Quality Initiative
e-GFR: Estimated glomerular filtration rate
